# Pharmacogenomic Approach to Identify Drug Sensitivity in Small-Cell Lung Cancer

**DOI:** 10.1371/journal.pone.0106784

**Published:** 2014-09-08

**Authors:** Gary Wildey, Yanwen Chen, Ian Lent, Lindsay Stetson, John Pink, Jill S. Barnholtz-Sloan, Afshin Dowlati

**Affiliations:** 1 Case Comprehensive Cancer Center, Case Western Reserve University, Cleveland, Ohio, United States of America; 2 Case Comprehensive Cancer Center and the Division of Hematology and Oncology, Case Western Reserve University, Cleveland, Ohio, United States of America; University of Colorado Denver, United States of America

## Abstract

There are currently no molecular targeted approaches to treat small-cell lung cancer (SCLC) similar to those used successfully against non-small-cell lung cancer. This failure is attributable to our inability to identify clinically-relevant subtypes of this disease. Thus, a more systematic approach to drug discovery for SCLC is needed. In this regard, two comprehensive studies recently published in *Nature,* the Cancer Cell Line Encyclopedia and the Cancer Genome Project, provide a wealth of data regarding the drug sensitivity and genomic profiles of many different types of cancer cells. In the present study we have mined these two studies for new therapeutic agents for SCLC and identified heat shock proteins, cyclin-dependent kinases and polo-like kinases (PLK) as attractive molecular targets with little current clinical trial activity in SCLC. Remarkably, our analyses demonstrated that most SCLC cell lines clustered into a single, predominant subgroup by either gene expression or CNV analyses, leading us to take a pharmacogenomic approach to identify subgroups of drug-sensitive SCLC cells. Using PLK inhibitors as an example, we identified and validated a gene signature for drug sensitivity in SCLC cell lines. This gene signature could distinguish subpopulations among human SCLC tumors, suggesting its potential clinical utility. Finally, circos plots were constructed to yield a comprehensive view of how transcriptional, copy number and mutational elements affect PLK sensitivity in SCLC cell lines. Taken together, this study outlines an approach to predict drug sensitivity in SCLC to novel targeted therapeutics.

## Introduction

Small cell lung cancer (SCLC) represents 15% of all lung carcinomas and is typically diagnosed when the disease has metastasized [1], [2]. Unfortunately there have been only minor improvements in the standard of care for SCLC over the past three decades [3]–[5]. There are currently no molecular targeted approaches to treat SCLC similar to those used successfully against non-small-cell lung cancer (NSCLC), such as erlotinib targeting of mutant EGFR or crizotinib targeting of EML4-ALK fusion proteins [6], [7]. Surgery is rarely performed in this disease (only 1% of cases), limiting the availability of tumor tissue for comprehensive genomic analyses. Furthermore, the two seminal genomics studies recently published on SCLC have yielded little therapeutic insight into this disease and have mainly analyzed the rare form of SCLC amenable to surgery, which does not represent the classic, widely metastatic SCLC seen in everyday clinical practice [8], [9].

A different approach to drug discovery for SCLC is needed and may lie in mining available databases on the drug sensitivities of SCLC cell lines. That is, as most SCLC cells are derived from metastatic sites or pleural effusions, they may be representative of extensive disease SCLC and its associated drug vulnerabilities. In this regard, two comprehensive drug-screening studies recently published in *Nature,* the Cancer Cell Line Encyclopedia (CCLE) [10] and the Cancer Genome Project (CGP) [11], examined the drug sensitivity of cancer cell lines, including lung, and attempted to link these to genomic profiles. The genomic profiles included DNA mutational status, gene expression and copy number variation (CNV) data.

In the present study we have specifically extracted the data on SCLC cell lines from these two studies and outline a bioinformatic approach to identify new therapeutics for SCLC using polo-like kinase (PLK) inhibitors as an example.

## Results

**Figure 1:**
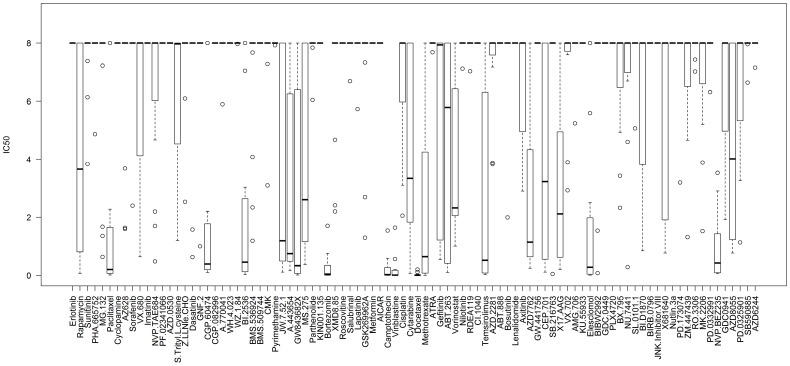
Boxplot of drug sensitivity in SCLC cells using the CGP dataset. There are 31 cell lines for small cell lung cancer. The boxplots show drugs listed on the x-axis and the corresponding IC_50_ values (in µM) listed on the y-axis. The ‘ceiling’ for drug efficacy was set at 8 µM; if the IC_50_ of all tested cells was above this concentration a single line would appear at the top of the graph. This represents an ineffective drug. By contrast, if all tested cells were sensitive to a given drug, a narrow box and whisker plot would appear at the bottom of the graph. The line within individual boxes represents the median IC_50_ value of all tested cells and the circles represent ‘outlier’ cells whose IC_50_ values do not fall within the 25–75% quantile of all IC_50_ values measured for that drug (represented by the box).

Initially we sought a global view of SCLC drug sensitivity in the CCLE [10] and CGP [11] studies. There were 53 and 31 SCLC cell lines tested for growth inhibition by 24 and 92 drugs in these studies, respectively. The results are shown in Figures 1 (CGP) and S1 (CCLE) as boxplots. A table of the numerical data for drug efficacy, as well as the outlier cell lines, is also given in Tables S1 (CGP) and S2 (CCLE). This graphical analysis allowed us to identify drugs that were broadly effective against most SCLC cells. We defined ‘effective’ drugs as those that induce growth inhibition in most cells at low doses (median IC_50_≤1 µM), represented by paclitaxel. ‘Ineffective’ drugs, represented by erlotinib and sunitinib, produced no growth inhibition in most SCLC cells (IC_50_≥8 µM), although ‘outliers’ may be present. ‘Selective’ drugs, represented by rapamycin, demonstrated a long boxplot and can be considered effective for only a subset of SCLC cell lines.

As shown in Table 1, drugs classified as ‘effective’ for most SCLC cells include CGP-60474, a CDK inhibitor; BI-2536 and GW-843682X, both PLK inhibitors; bortezomib, a proteasome inhibitor; and elesclomol, an HSP70 inhibitor. In addition, several drugs targeting the PI3K-AKT-MTOR pathway fall within this category, including A-443654, temsirolimus and NVP-BEZ235. Two drugs with a median IC_50_ just outside 1 µM include AZD-7762, a CHK inhibitor; and JW-7-52-1, an MTOR inhibitor. HSP90 (17-AAG) and HDAC (panobinostat) inhibitors may also represent ‘effective’ drugs, although their efficacy varied among the two studies. ‘Effective’ drugs likely carry the best translational potential.

**Table 1:** ‘Effective’ drugs on SCLC cells.

	Quantile (µM)			
Drug	25%	50%	75%	Target
**CCLE:**				
17-AAG*	0.042	0.19	0.5	HSP90
Panobinostat	0.02	0.05	0.07	HDAC
**CGP:**				
CGP.60474	0.2	0.39	1.77	CDK1/2/5/7/9
BI-2536	0.13	0.46	2.64	PLK1/2/3
Bortezomib	0.01	0.04	0.35	PSMB5
Elesclomol	0.04	0.28	1.98	HSP70
NVP.BEZ235	0.09	0.43	1.43	PI3K or MTORC1/2
A.443654	0.49	0.75	6.25	AKT1/2/3
GW843682X	0.07	0.33	6.39	PLK1
Temsirolimus	0.08	0.52	6.31	MTOR
AZD7762	0.64	1.15	4.33	CHK1/2
X17.AAG*	0.62	2.11	4.94	HSP90
JW.7.52.1	0.49	1.19	8	MTOR
MS.275	1.16	2.61	8	HDAC
Vorinostat	20.5	2.32	6.43	HDAC

*overlapping drug between two studies

**Figure 2:**
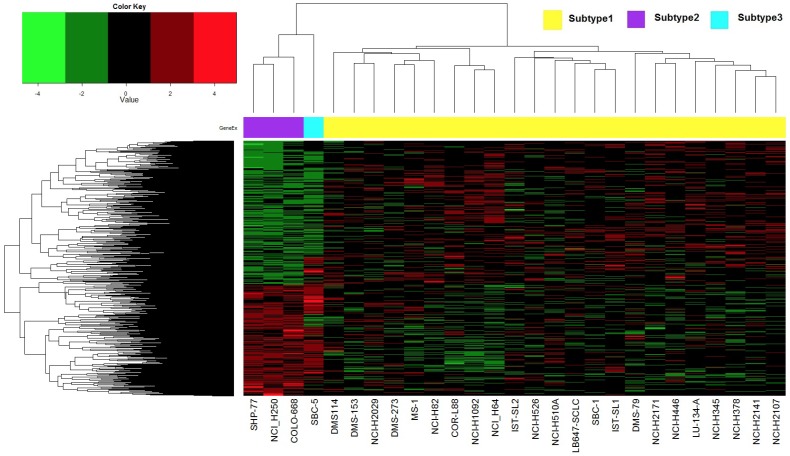
Unsupervised clustering of SCLC cells by gene expression using the CGP dataset. Unsupervised consensus clustering was performed using all 31 cell lines (only 27 had gene expression data available; 3 of them are duplicates and the average values were obtained for further analysis) and showed that 3 clusters was optimal for this dataset. With this assignment, non-parametric one way ANOVA (Kruskal-Wallis test p-value<0.05) was performed on these 3 clusters and 1006 significant genes were obtained. The heatmap was generated with these significant genes.

**Figure 3:**
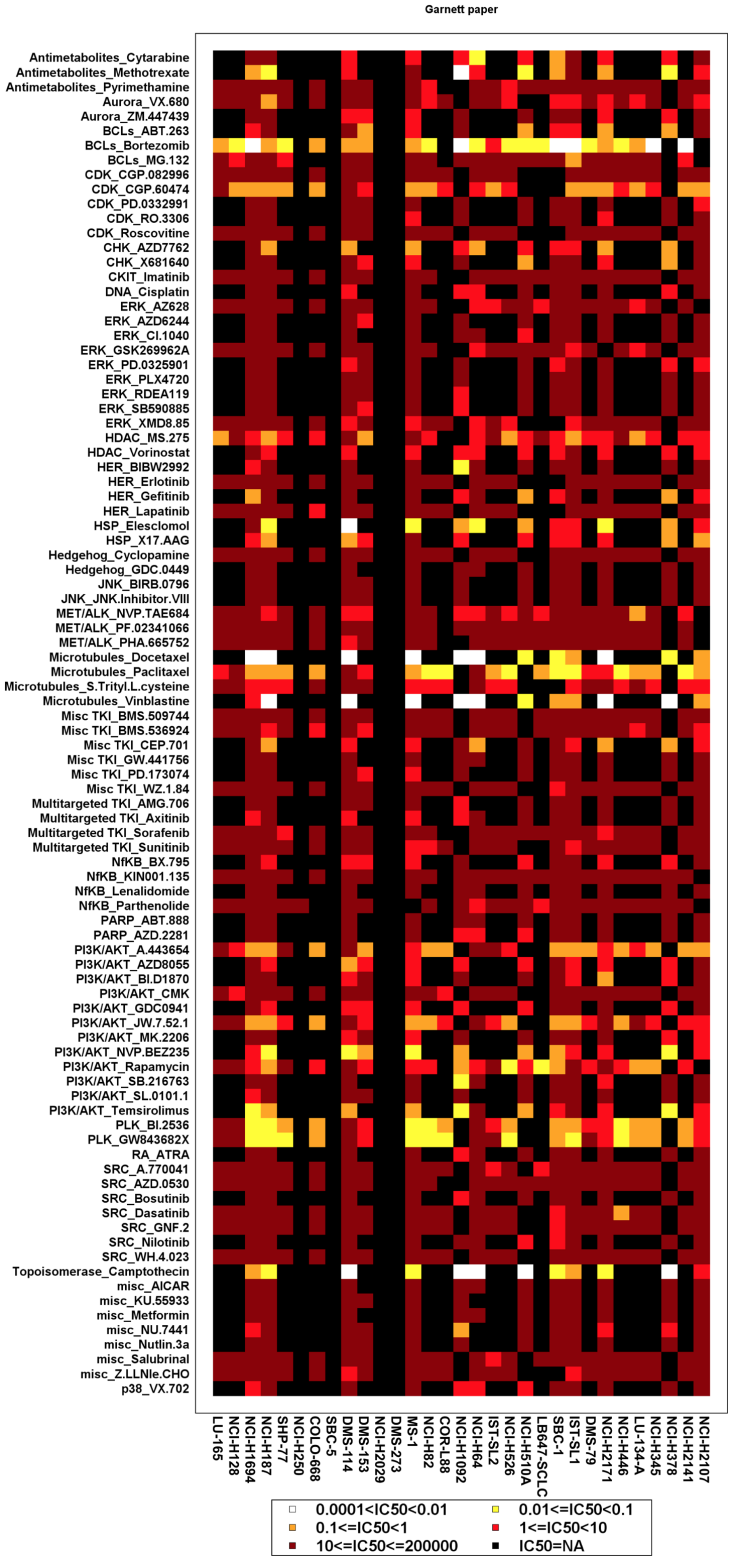
Mosaic plot of drug sensitivity using gene expression clustering of SCLC cells. Drug sensitivity was color-coded according to the legend at the bottom. Drugs are grouped along the y-axis according to their target molecule. Cells are arranged along the x-axis identical to their gene expression clustering identified in Figure 2.

Gene array data has been used extensively in cancer research to identify expression ‘signatures’ that may be either prognostic or predictive of tumor behavior. Therefore, we examined if gene expression clustering could be used to identify subgroups of drug-sensitive SCLC cell lines, particularly for drugs that demonstrated a broad efficacy range against SCLC cells in Figure 1. We used data from the CGP study because it contained the largest amount of drug sensitivity data. Unsupervised consensus clustering of gene expression data demonstrated that three clusters of SCLC cells were optimal (Figure 2). There were 1006 significant genes that defined the gene expression subtypes (Kruskal-Wallis test p-value <0.05, listed in Table S3. We then visualized the effect of gene expression clustering on drug efficacy using a mosaic plot (Figure 3). In this plot we used a color scale that divided drug sensitivity into six groups. Drugs, listed on the y-axis, were grouped together according to target molecules. Cells, listed on the x-axis, were grouped using the same order as that obtained by unsupervised clustering of their gene expression. There did not appear to be any correlation of drug sensitivity to gene expression clustering, however, as drug sensitivity appeared to be randomly distributed across all cell lines for any given drug. This graphical analysis did highlight several targeted agents with exceptional broad-based efficacy against SCLC cell lines. These drugs included bortezomib, BI-2536 and GW-843682X, as well as the HSP inhibitor elesclomol and the CDK inhibitor CGP-60474, albeit with lower efficacy. These drugs are identical to those highlighted in Table 1.

**Figure 4:**
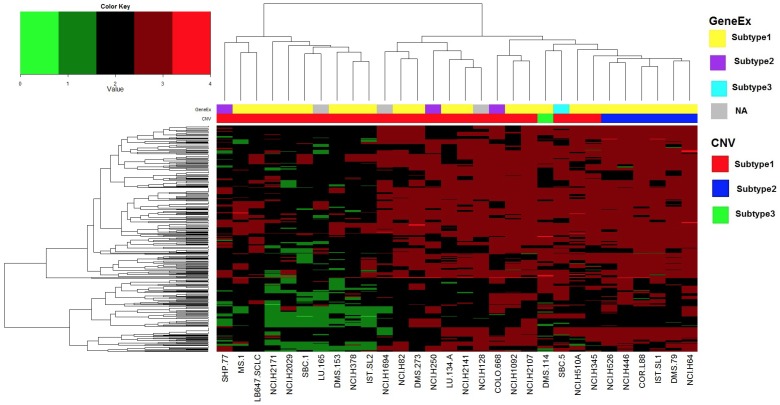
Unsupervised clustering of SCLC cells by copy number variation using the CGP dataset. Unsupervised consensus clustering was performed using all 30 cell lines with 426 gene copy numbers, and 3 clusters were shown to be optimal for this dataset. All of these 426 genes were used to generate the heatmap. CNV data was re-coded according to the following rule: 0-complete loss; 1-partial loss; 2-no change; 3∼7-partial gain; greater or equal to 8-complete gain.

We next determined if CNV clustering could be used to identify subgroups of drug-sensitive SCLC cell lines. Three clusters were again identified using all 426 interrogated genes (Figure 4); however there did not appear to be any correlation between the gene expression and CNV clustering. Rearrangement of the mosaic plot in Figure 3 by CNV clustering also did not reveal any apparent correlations (Figure S2). Taken together, these results demonstrate that drug-sensitive SCLC cells did not cluster into subgroups by either gene expression or CNV. We therefore decided to use a pharmacogenomic approach to characterize subgroups of SCLC cells.

**Figure 5:**
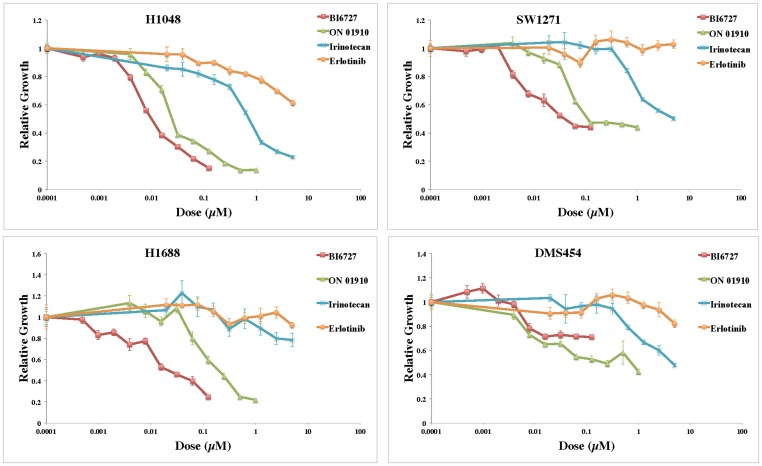
Validation of efficacy of PLK inhibitors in SCLC cells. Adherent cells were incubated with the indicated concentrations of drugs for 24 h. The cell culture medium was replaced and cell viability was measured by a DNA assay after 48 h incubation. Each drug concentration was assayed utilizing five replicates. Results are representative of at least 2 experiments.

Our analyses identified PLK inhibition to have promising efficacy and little clinical trial activity in SCLC. Therefore, we used PLK inhibition as an example of how to use the CGP datasets to develop a genomic profile of SCLC drug sensitivity. First we sought to validate the efficacy of PLK inhibitors in SCLC. In these validation experiments we used SCLC cell lines, as well as inhibitors, that were different from those used in the original studies to highlight the efficacy of these new therapeutic agents. The SCLC cell lines used were H1048, H1688, SW1271 and DMS454. PLK inhibitors included BI-6727 (volasertib) and ON-01910 (rigosertib). In these experiments irinotecan served as a positive control while erlotinib served as a negative control. The results are shown in Figure 5. It is clear that the IC_50_ values for the PLK inhibitors in most cell lines is between 10-100 nM, supporting our hypothesis that SCLC cells are broadly sensitive to PLK inhibitors.

**Figure 6:**
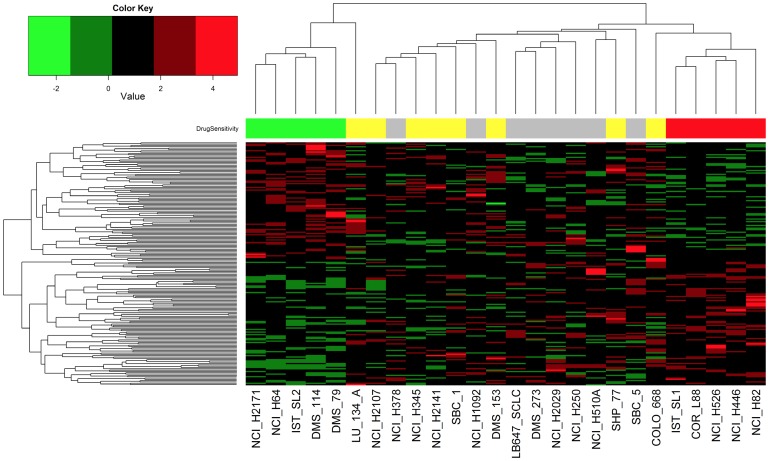
Unsupervised clustering of SCLC cells using the PLK gene signature. The five SCLC cell lines demonstrating the most (H2171, H64, IST-SL2, DMS-114, DMS-79) and least (IST-SL1, COR-L88, H526, H446, H82) resistance to the PLK inhibitor BI-2536 in the CGP study were used as standards to identify a gene signature for PLK sensitivity. All SCLC cell lines in the CGP study that contained gene expression data were then subjected to unsupervised clustering. The heatmap shows the result of this analysis. The colored boxes on the top of the heatmap indicate the CGP BI-2536 sensitivity. Green  =  resistant cell, red  =  sensitive cell, yellow  =  cell of intermediate, but known, sensitivity, grey  =  cell of untested sensitivity but with gene expression data.

Initially, we sought to identify a gene signature that might predict sensitivity to PLK inhibitors in patient cohorts, as not all SCLC cells demonstrated equal sensitivity. We compared the gene expression data for the five most sensitive SCLC cells (H82, H446, H526, COR L88, IST SL1) with that for the five least sensitive SCLC cell lines (DMS114, H64, DMS79, H2171, IST SL2); as defined in the CGP by their BI-2536 IC_50_ values. We identified a list of 185 genes that were significantly differentially expressed between these two groups (listed in Table S4). These 185 genes were used to perform unsupervised clustering of all the SCLC cell lines, resulting in the heatmap shown in Figure 6. Notably, the five least (green top box) and most (red top box) sensitive cell lines clustered at opposite ends of the heatmap while all the other cells (yellow boxes indicating intermediate sensitivity and grey boxes indicating unknown sensitivity) clustered in the middle. We next performed leave-one-out analysis of the PLK gene signature with the 26 available cell lines. From this analysis we generated 26 heatmaps, where in each heatmap the sensitive and resistant cells remained clustered together except in three heatmaps; in which one or two sensitive cell lines were misclassified. Resistant cells always clustered together. Hence, we believe that this PLK gene signature is robust in categorizing sensitive and resistant SCLC cell lines.

**Figure 7:**
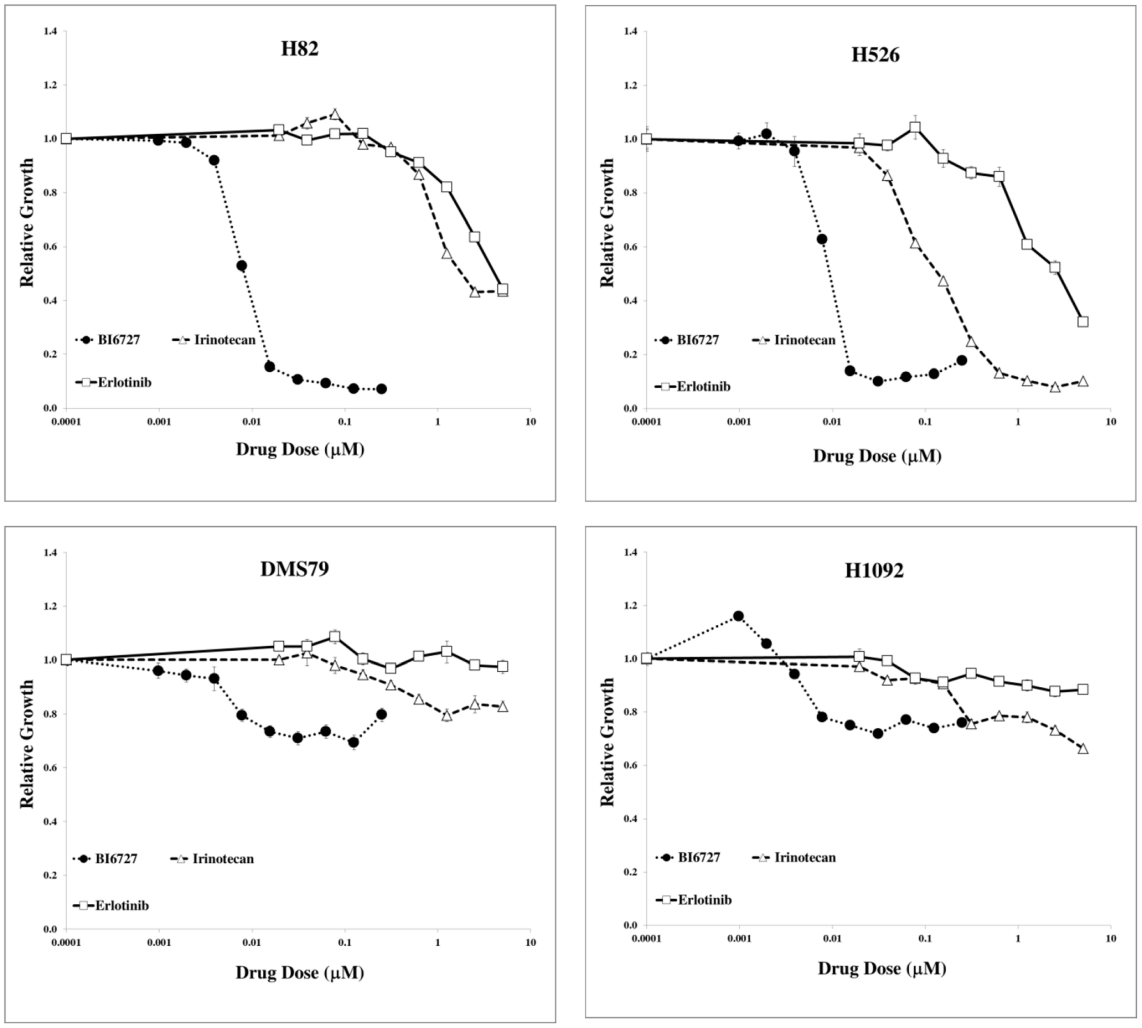
Validation of PLK efficacy in SCLC cells predicted by PLK gene signature. Suspension cells were continuously incubated with the indicated concentrations of drugs for 72 h, when cell viability was measured by the MTS assay. Each drug concentration was assayed utilizing five replicates. Results are representative of 2 experiments.

To validate that the PLK gene signature does, in fact, predict sensitivity to PLK inhibitors, we determined the efficacy of the PLK inhibitor BI-6727 in a cell line, H1092, which had gene expression data but no PLK sensitivity data in the CGP study, represented by a grey box at the top of Figure 6. As controls, we used one resistant cell line, DMS79 (green box), and two sensitive cell lines, H82 and H526 (red boxes). These cells were chosen because they all grew in suspension and could be subjected to identical drug treatment protocols. The results, shown in Figure 7, demonstrated that H82 and H526 cells were sensitive to the PLK inhibitor BI-6727 whereas DMS79 cells were not, similar to the results reported for BI-2536. Furthermore, it demonstrated that the H1092 cells, with unknown PLK sensitivity, were mostly resistant to BI-6727 like DMS79 cells, as predicted by the heatmap.

**Figure 8:**
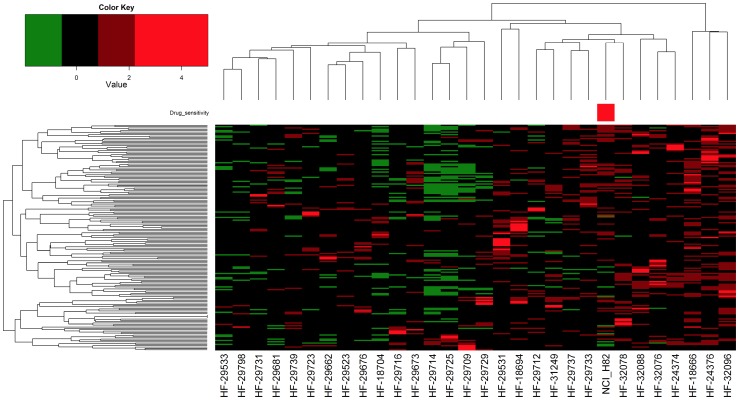
Supervised clustering of SCLC tumors and H82 cell line using the PLK gene signature. RNAseq data from Rudin et al. [8] was transformed to count data. Data for genes that comprised the PLK gene expression signature were extracted and used in unsupervised consensus clustering of SCLC tumors and the H82 cell line. The red colored box on the top of the heatmap indicates the location of the H82 cell line, which was a CGP cell line validated as PLK sensitive.

It was of interest to determine whether the PLK gene signature was present in patient tumors and could potentially be used to predict tumor sensitivity to PLK inhibitors. The largest study of SCLC tumor gene expression is that of Rudin et al. [8], who analyzed 30 primary tumors by RNAseq. We therefore extracted the count data for the 185 probes present in our PLK signature (representing 173 genes; only 169 genes were found and used from the Rudin dataset) and standard normalized to create surrogate PLK gene expression arrays for these tumors. This normalized data was then subjected to unsupervised clustering to generate the heatmap shown in Figure 8. We also included in this analysis the H82 SCLC cell line that had RNAseq data from the Rudin study and was also validated by us in Figure 7 as being sensitive to the PLK inhibitor BI-6727. The results demonstrate two important points: first, subtypes of SCLC tumors can be identified using the PLK gene signature, and second, the H82 cell line data clustered among a subset of eight primary tumors. Taken together, these results suggest that the PLK gene signature generated using SCLC cell line data may be useful in predicting the sensitivity of specific tumors to PLK inhibitors.

**Figure 9:**
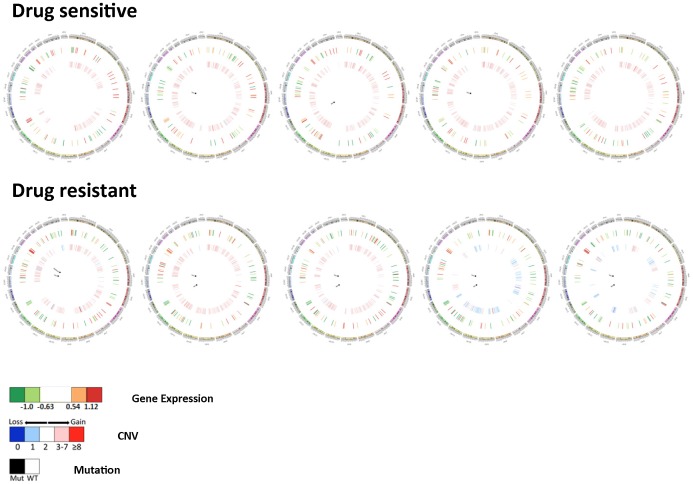
Circos plots of SCLC cells. Circos plots are shown (left to right, in listed order) for the five most sensitive (H82, H446, H526, COR-L88, IST-SL1) and most resistant (DMS-114, H64, DMS-79, H2171, IST-SL2) SCLC cells to BI-2536 growth inhibition, as defined in the CGP study. The outer black ring designates the chromosome location; the next inner ring indicates expression level of PLK signature genes; and the inner most ring indicates CNV. CNV data was re-coded according to the following rule: 0 complete loss; 1 partial loss; 2 no change; 3∼7 partial gain; greater or equal to 8 complete gain. At the center is the mutation status of genes (open triangle: intronic SNP, black circle: missense SNP, black square: nonsense SNP, red triangle: frame-shift insertion, green triangle: frame-shift deletion). All mutations were homozygous except for the frame-shift insertion.

Finally, we used circos plots [12], shown condensed in Figure 9 and full-view in Figure S3, to visualize the genomic differences between SCLC cell lines sensitive and resistant to the PLK inhibitor BI-2536. Remarkably, the circos plots demonstrate that all resistant cells possessed nonsense or frameshift mutations in either *TP53* or *RB1*, and sometimes both genes, whereas all sensitive cells displayed mutations of unknown protein significance (intronic and missense), typically in only one of these genes. All but one of the gene mutations was homozygous, indicating that resistant cells likely have no functional RB1 or TP53 protein. All sensitive cells display *MYC* (H82, H446), *MYCN* (H526, IST SL1) or *MYCL* (CORL88) amplification, whereas only one resistant cell line displays *MYC* amplification (H2171). There also seems to be little or no CNV on the X chromosome in sensitive cells relative to resistant cells, which typically display some CNV loss. Genes on chromosome 13 are generally upregulated in PLK sensitive cells and downregulated in PLK resistant cells, whereas genes located on chromosome 19 are generally downregulated in PLK sensitive cells and upregulated in PLK resistant cells. Taken together, these results suggest that gene expression, CNV and mutational status may all contribute to the sensitivity of SCLC cells to PLK inhibition.

## Discussion

Small-cell lung cancer is a disease in urgent need of new drug therapies. Unfortunately, the limited availability of tumor tissue hinders the acquisition of complete genomic analyses required for the identification and validation of new drugable targets in this cancer. Thus, alternative drug discovery strategies need to be developed until our understanding of the genomics driving SCLC can begin to approximate that of NSCLC, which benefits from comprehensive analyses of large cohorts of patient tumors, such as the The Cancer Genome Atlas (TCGA).

In this report we have taken a bioinformatic approach to drug discovery for SCLC by data mining two large drug screening studies in cultured cell lines, the CCLE [10] and CGP [11]. As a model system, SCLC cell lines retain gene mutation profiles (COSMIC) and copy number changes [13], [14] similar to human SCLC. Therefore, we have extracted and analyzed datasets for SCLC cell lines in order to identify drug sensitivities specific to this disease, as this was not the intention of the original studies, which was to pool data across a multitude of cell lines in order to identify genomic determinants of drug sensitivity. We identified polo-like kinases as attractive molecular targets with little current clinical trial experience in SCLC. Growth inhibition by PLK inhibitors was validated in our study, addressing concerns raised in a recent report about inconsistency in drug response data in large drug screening studies [15]. The translational potential of PLK inhibitors in treating SCLC is supported by our demonstration that the drug sensitivity profile of the SCLC cell lines reflects what is observed clinically for metastatic SCLC tumors [16]. That is, most cells were extremely sensitive to topoisomerase and microtubule inhibitors, chemotherapeutic agents that have activity against chemo-naïve SCLC; by contrast, many tyrosine kinase inhibitors were ineffective.

The CGP study also identified drug sensitivities that tended to cluster across all cell lines (see Supplement Table 1 in reference 11) and the drug sensitivity profile for SCLC cells, as shown in Table 1, is very similar to cluster 4 of the CGP study [cluster 4  =  GW-843682X (PLK1), BI-2536 (PLK1/2/3), A-443654 (AKT1/2/3), Epothilone B (microtubules), CGP-60474 (CDK1/2/5/7/9), Paclitaxel (microtubules) and MS-275 (HDAC)]. While it is currently unclear if these drug clusters indicate a common targeted pathway(s) leading to growth inhibition, these clusters may provide a practical starting point to test combinatorial drug therapies for synergistic activity in SCLC. Indeed, our own preliminary data shows synergism between PLK and CDK inhibitors.

We have developed a highly integrated analysis of PLK sensitivity in SCLC cell lines, graphically depicted in Figure 9 as circos plots, that incorporates gene expression, CNV and gene mutation data. An analysis of this depth has never been previously applied to SCLC, and demonstrates what can be achieved interrogating a single cell lineage and drug class in the CCLE and CGP datasets. Furthermore, our finding that the PLK gene signature for SCLC cell lines was dispersed among SCLC tumor specimen expression profiles (Figure 8) clearly demonstrates that these cells retain tumor phenotypes. Features in the circos plots such as the double mutation of both *TP53* and *RB1* in PLK resistant cells, as well as the reciprocal expression of PLK signature genes on chromosomes 13 and 19, are readily apparent and must be examined in larger cohorts to determine their individual contribution to overall PLK inhibitor sensitivity. Taken together, this type of analysis may help to identify upstream genomic events that correlate with downstream phenotypes such as PLK sensitivity.

It was recently reported that mutations in the *PLK1* gene itself were primarily responsible for acquired resistance to BI2536 in a cultured human colon cancer cell line [17]. This is unlikely to be a resistance mechanism in SCLC cell lines because the CCLE lists only four cell lines with a single *PLK* mutation among all four PLK family members (PLK1-4) and 53 SCLC cell lines examined. None of these PLK mutated cell lines were included in our study. Our PLK gene signature does, however, include genes on chromosomes typically deleted (4q, 13q) and amplified (19p) in SCLC [9], [13], [14], although our circos plots reveal no obvious correlation between the two. Interestingly, chromosome Xq, which is not typically viewed as an important region of CNV in SCLC, was home to several of the most significant differentially-expressed genes that comprise the PLK gene signature- all were members of the MAGE-A, or melanoma-associated antigen-A, subfamily. This subfamily of genes is located on chromosome Xq28 and is only expressed in testis germ cells and tumor cells [18]. Although their biologic function is unclear, they represent a class of tumor antigens that are being actively investigated as a target for immunotherapy [19], [20]. The MAGE-A genes were upregulated in PLK-sensitive cell lines relative to PLK-insensitive cell lines. Furthermore, these expression patterns may correlate with CNV, as sensitive cells demonstrated little CNV on chromosome X, whereas most resistant cells demonstrated some CNV loss. We are currently testing the importance of this gene family as a biomarker of PLK sensitivity.

During our study a report by Sos et al. [21] was published in PNAS that specifically surveyed only SCLC cell lines (44 total) for drug sensitivity. Of the 267 compounds tested in this study, only 13 were also examined in the CCLE and/or CGP studies. Interestingly, when Sos et al. looked for drug sensitivity specifically in *MYC*-amplified cell lines, they also found the PLK inhibitor BI-2536 to be active, similar to our results, but did not pursue it further. They also showed that the majority of cells sensitive to the Aurora kinase inhibitor VX-680 demonstrated *MYC*-amplification. Interestingly, the CGP also included two Aurora kinase inhibitors (VX-680 and ZM-447439) in their study; however, it did not find any significant clustering of PLK with Aurora kinase inhibitors, indicating no link between these two classes of inhibitors when analyzed in the general population of cancer cell lines.

In the present study we consistently identified three subtypes of SCLC cell lines using unsupervised clustering of either gene expression or CNV datasets from the CGP or CCLE (Figure S4 and Table S3) studies. Remarkably, there was little concordance between these two analyses except that a great majority of cells clustered into one predominant subtype, while the remaining cells divided unequally between two minor subtypes. Gene expression and CNV subtypes also did not align with drug sensitivity in general (see Figures 3 and S2) or PLK sensitivity in particular. This suggests that a comprehensive genomics approach, such as circos plot analysis, is required to identify critical determinants of drug sensitivity and other phenotypes in SCLC. This integrated approach may help to select SCLC patients that would benefit most from single agent use of drugs such as HDAC [22] and PLK [23] inhibitors that are broadly effective across SCLC cell lines but demonstrate limited activity in clinical trials.

Other unique approaches have been taken to identify new and effective therapies for SCLC. Jahchan et al. identified a surprising sensitivity of SCLC cells to tricyclic antidepressants in a drug repositioning study, which used bioinformatics to find drugs that induced changes in gene expression opposite to the gene expression profile of SCLC cells [24]. Reverse-phase protein arrays (RPPA) were used to identify PARP1 and EZH2 as potential therapeutic targets in SCLC [25]. Ultimately, there is a need to reveal the underlying biology of SCLC if we hope to make any improvements in the treatment of this disease similar to NSCLC. Unfortunately, only about fifty SCLC tumors have undergone comprehensive genomic analysis to date- the majority being from primary, early stage disease [8], [9]. Therefore, immediate therapeutic progress in this field will depend upon discovery in model systems, such as the one outlined here, followed by validation in patient cohorts.

## Materials and Methods

### Cell culture and growth inhibition studies

All cells were obtained from the ATCC and grown in their recommended medium. Cell proliferation was determined quantitatively by fluorescent DNA assay [26] for adherent cells or MTS assay using the CellTiter 96 AQueous One Solution Cell Proliferation Assay Kit from Promega Corp. (Fitchburg, WI) for suspension cells. Cells were added to a 96-well plate in 100 µl of complete medium. Drug containing medium was added at the indicated concentrations one day after seeding. Irinotecan was obtained from Sigma Chemical Co. (St. Louis, MO) while all other drugs were obtained from Selleck Chemicals (Houston, TX). After incubation for three days at 37°C, the assay was performed following the manufacturer’s instructions. For the DNA assay, fluorescence was measured using 355/460 nm excitation/emission filters while absorbance for the MTS assay was measured at 490 nm. Both assays were measured with a 96-well plate reader. Each experimental condition was assayed utilizing five replicates. Drug containing medium was removed from adherent cells after 24 h of incubation and replaced with 100 µl of complete medium, while suspension cells were grown in the continuous presence of drug for 72 h.

### Datasets

The publically available CCLE and CGP drug sensitivity (IC_50_), gene expression, CNV, and mutation data was downloaded from http://broadinstitute.org/ccle (CCLE) and http://cancerrxgene.org (CGP) [10], [11]. The RNAseq data obtained in the study by Rudin et al. [8] was downloaded from the European Genome database. All data manipulation and statistical analyses were performed using SAS version 9.3 (SAS Institute Inc., Cary, NC) or R 2.15.3 (http://www.r-project.org/).

### IC_50_ data analysis

For SCLC only, 24 drugs and 53 cell lines were included in the CCLE, while 92 drugs and 31 cell lines were included in the CGP. All IC_50_ greater than 8 µM in the CGP were thresholded at 8 µM. Boxplots were drawn for the IC_50_ from the two datasets respectively using R (http://www.r-project.org/). Mosaic plots were drawn using proc sgrender in SAS version 9.3 (SAS Institute Inc., Cary, NC).

### Gene expression data analysis

Normalization of gene expression data (Affymetrix U133 Plus 2.0 for CCLE, Affymetrix U133A for CGP) involved four steps: 1) raw data were normalized via the robust multi-array average (RMA) method; 2) probes without a gene name were removed; 3) gene level data was obtained by averaging the probe value within each gene and 4) the gene level data was then standard normalized by gene. For the gene expression data, the CCLE included 51 cell lines, while the CGP included 30 cell lines, among which three cell lines had duplicate measurements. Averaging the duplicates generated 27 unique cell lines for the CGP. Unsupervised consensus clustering was performed on the normalized gene level data with the R package “Consensus Cluster Plus”; parameters were set as default except maxK was set at 10. The Kruskal-Wallis test was used to assess expression differences between subtypes based on the consensus clustering results. Unsupervised hierarchical clustering was then performed using the significant genes only (p value <0.05) and visualized using heatmaps via the R package “gplots”.

### Gene copy number data analysis

Copy number data was obtained for the CGP data only for 426 genes [11]. Generation of subtypes based on the raw copy number data was performed using unsupervised consensus clustering. CNV raw data from CGP was re-coded according to the following rule: 0 complete loss; 1 partial loss; 2 no change; 3∼7 partial gain; greater or equal to 8 complete gain. Visualization via heatmaps was performed with transformed copy number data using the same algorithms as described in the gene expression data analysis section.

### RNAseq analysis

The fastq data was aligned with tophat 2.0.9 [27], followed by HTseq 0.5.4 [28] in order to obtain the count data. The count data was then standard normalized by gene for tumor data or cell line data, respectively. The initial PLK gene signature analysis identified 185 probes in 173 genes; only 169 of the genes were found in the Rudin RNAseq data. Unsupervised clustering using this set of 169 genes was performed on the combined tumor and H82 cell line data.

## Supporting Information

Figure S1 **Boxplot of drug sensitivity in SCLC cells using the CCLE dataset.** There are 53 cell lines for small cell lung cancer. The boxplots show drugs listed on the x-axis and the corresponding IC_50_ values (in µM) listed on the y-axis, similar to Figure 1.
(PDF)

Figure S2 **Mosaic plot of drug sensitivity using CNV clustering of SCLC cells.** Drug sensitivity was color-coded according to the legend at the bottom. Drugs are grouped along the y-axis according to their target molecule. Cells are arranged along the x-axis identical to their gene expression clustering identified in Figure 4.
(TIF)

Figure S3 **Full-size circos plots of SCLC cells.** Circos plots are shown full-size as individual panels for all the SCLC cell lines shown in collectively Figure 9. The drug sensitivity (PLK sensitive vs resistant) of the individual cells is indicated on the left, along with the legend. The gene mutation symbols are identical to those described for Figure 9.
(PPTX)

Figure S4 **Unsupervised clustering of SCLC cells by gene expression using the CCLE dataset.** Unsupervised consensus clustering was performed using the all 53 cell lines (only 51 gene expression available) and showed that 3 clusters was optimal for this dataset. With this assignment, we performed non-parametric one way ANOVA (Kruskal-Wallis) test on those 3 clusters and obtained 4749 significant genes. We then generated the heatmap with those significant genes.
(TIF)

Table S1 **Numerical data for drug efficacy determined in the CGP study.** The 25%, 50% and 75% quantiles for all 92 drugs used to construct the boxplot in Figure 1 are listed. The outlier cell lines with IC50s <4 µM are listed to the right (IC50s of outliers in parentheses in µM).
(DOC)

Table S2 **Numerical data for drug efficacy determined in the CCLE study.** The 25%, 50% and 75% quantiles for all 24 drugs used to construct the boxplot in Figure S1 are listed. The outlier cell lines with IC50s <4 µM are listed to the right (IC50s of outliers in parentheses in µM).
(DOC)

Table S3 **Significant genes used in CGP and CCLE gene expression clustering.** The 1006 and 4749 significant genes used to cluster SCLC cell lines in the CGP (Figure 2) and CCLE (Figure S4) datasets, respectively, are highlighted along with their corresponding p-values.
(XLS)

Table S4 **Significant genes used in PLK gene expression clustering.** The 185 significant genes used to cluster SCLC cell lines based upon their sensitivity to BI-2536 (Figure 6) using the CGP dataset are highlighted along with their corresponding p-values.
(XLS)
