# Supplementary material for: Pharmacogenomic Approach to Identify Drug Sensitivity in Small-Cell Lung Cancer
Source: PLoS One. 2014 Sep 8;9(9):e106784. doi: 10.1371/journal.pone.0106784 (PMC4157793; doi:10.1371/journal.pone.0106784)

Figure S1. CCLE Boxplot and data for IC50s: There are 53 cell lines for small cell lung cancer. The boxplots show drugs listed on the x-axis and the corresponding IC_{50} values (in μM) listed on the y-axis. The 'ceiling' for drug efficacy was set at 8 μM; if the IC_{50} of all tested cells was above this concentration a single line would appear at the top of the graph. This represents an ineffective drug. By contrast, if all tested cells were sensitive to a given drug, a narrow box and whisker plot would appear at the bottom of the graph. The line within individual boxes represents the median IC_{50} value of all tested cells and the circles represent 'outlier' cells whose IC_{50} values do not fall within the 25-75% quantile of all IC_{50} values measured for that drug (represented by the box).

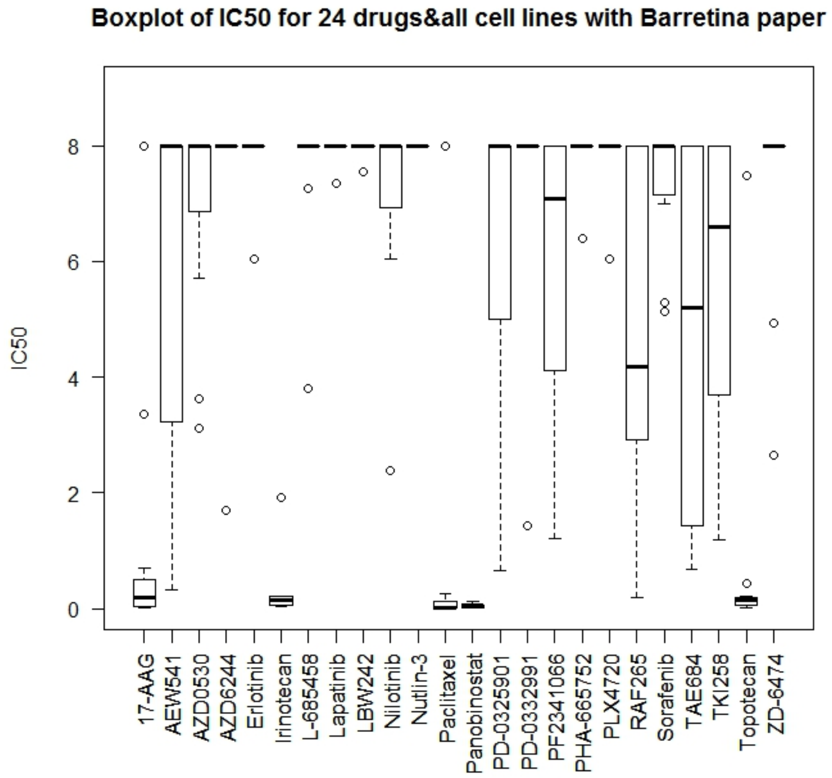

Supplement: Figure S1 — Boxplot of drug sensitivity in SCLC cells using the CCLE dataset. There are 53 cell lines for small cell lung cancer. The boxplots show drugs listed on the x-axis and the corresponding IC50 values (in µM) listed on the y-axis, similar to Figure 1. (PDF) [file pone.0106784.s001.pdf]
