# Supplementary material for: Pharmacogenomic Approach to Identify Drug Sensitivity in Small-Cell Lung Cancer
Source: PLoS One. 2014 Sep 8;9(9):e106784. doi: 10.1371/journal.pone.0106784 (PMC4157793; doi:10.1371/journal.pone.0106784)
Supplement: Figure S3 — Full-size circos plots of SCLC cells. Circos plots are shown full-size as individual panels for all the SCLC cell lines shown in collectively Figure 9. The drug sensitivity (PLK sensitive vs resistant) of the individual cells is indicated on the left, along with the legend. The gene mutation symbols are identical to those described for Figure 9. (PPTX) [file pone.0106784.s003.pptx]

## Slide 1
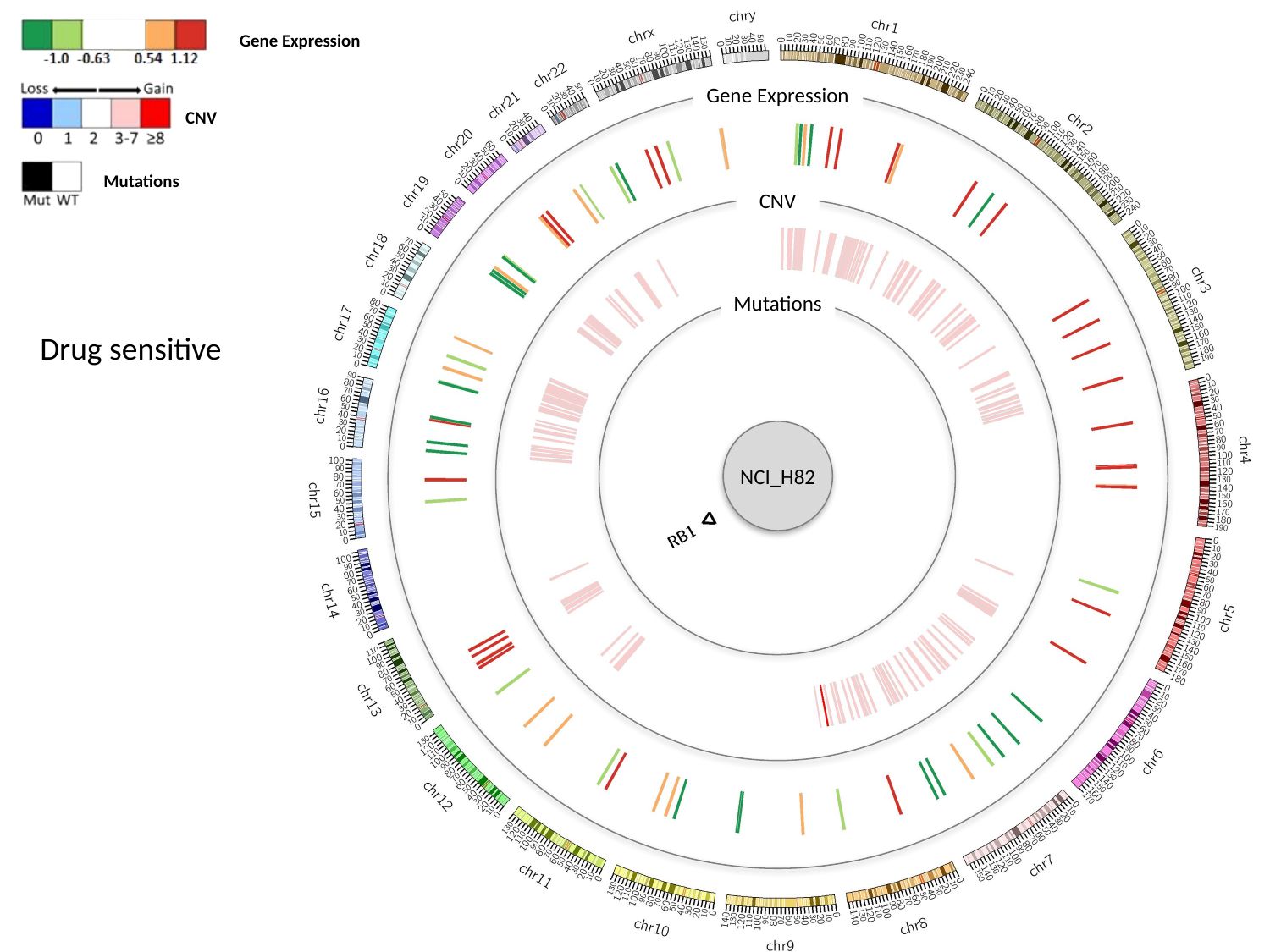

Gene Expression
CNV
Mutations
NCI_H82
Gene Expression
CNV
Mutations
Drug sensitive

## Slide 2
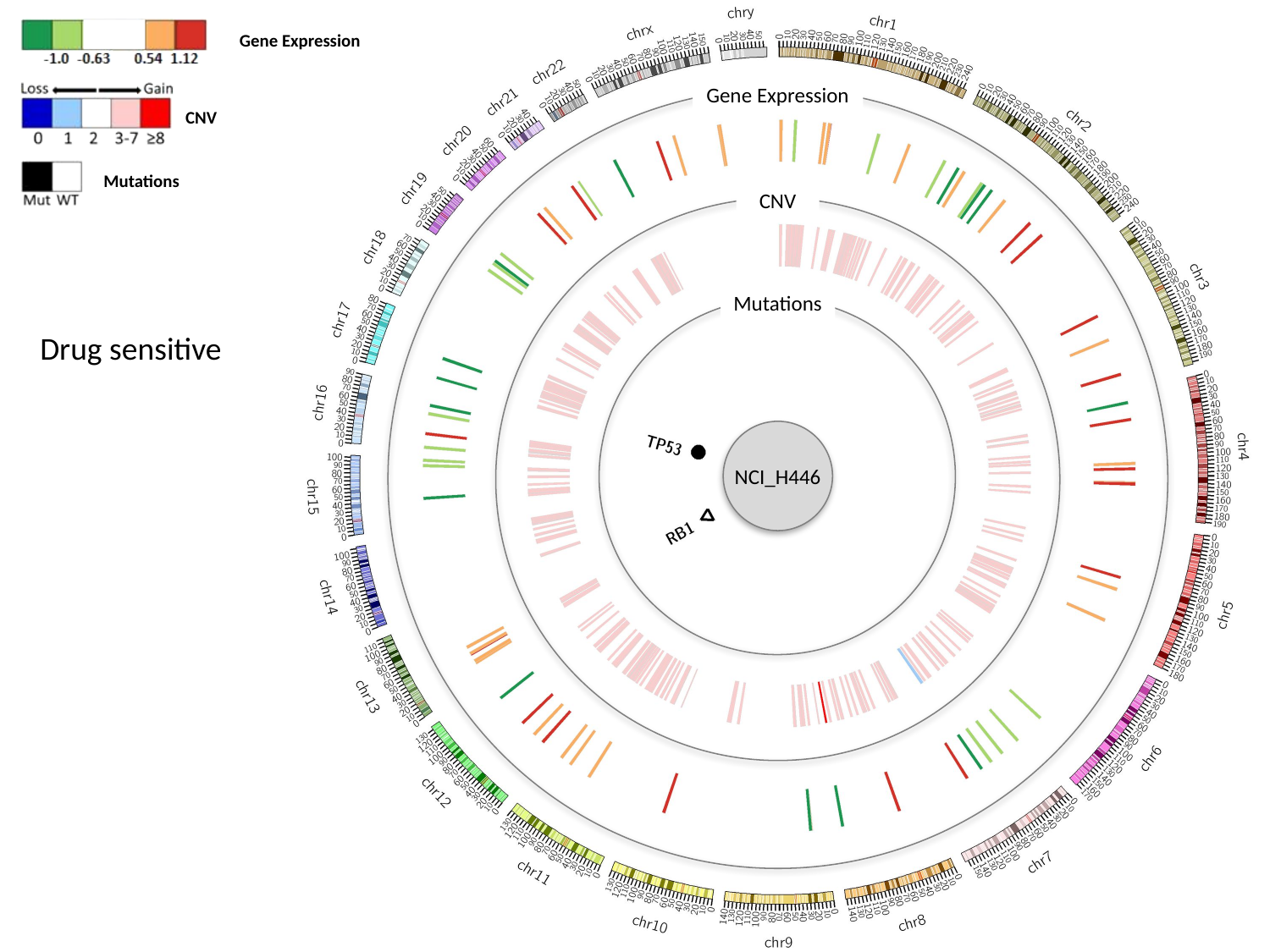

Gene Expression
CNV
Mutations
NCI_H446
Gene Expression
CNV
Mutations
Drug sensitive

## Slide 3
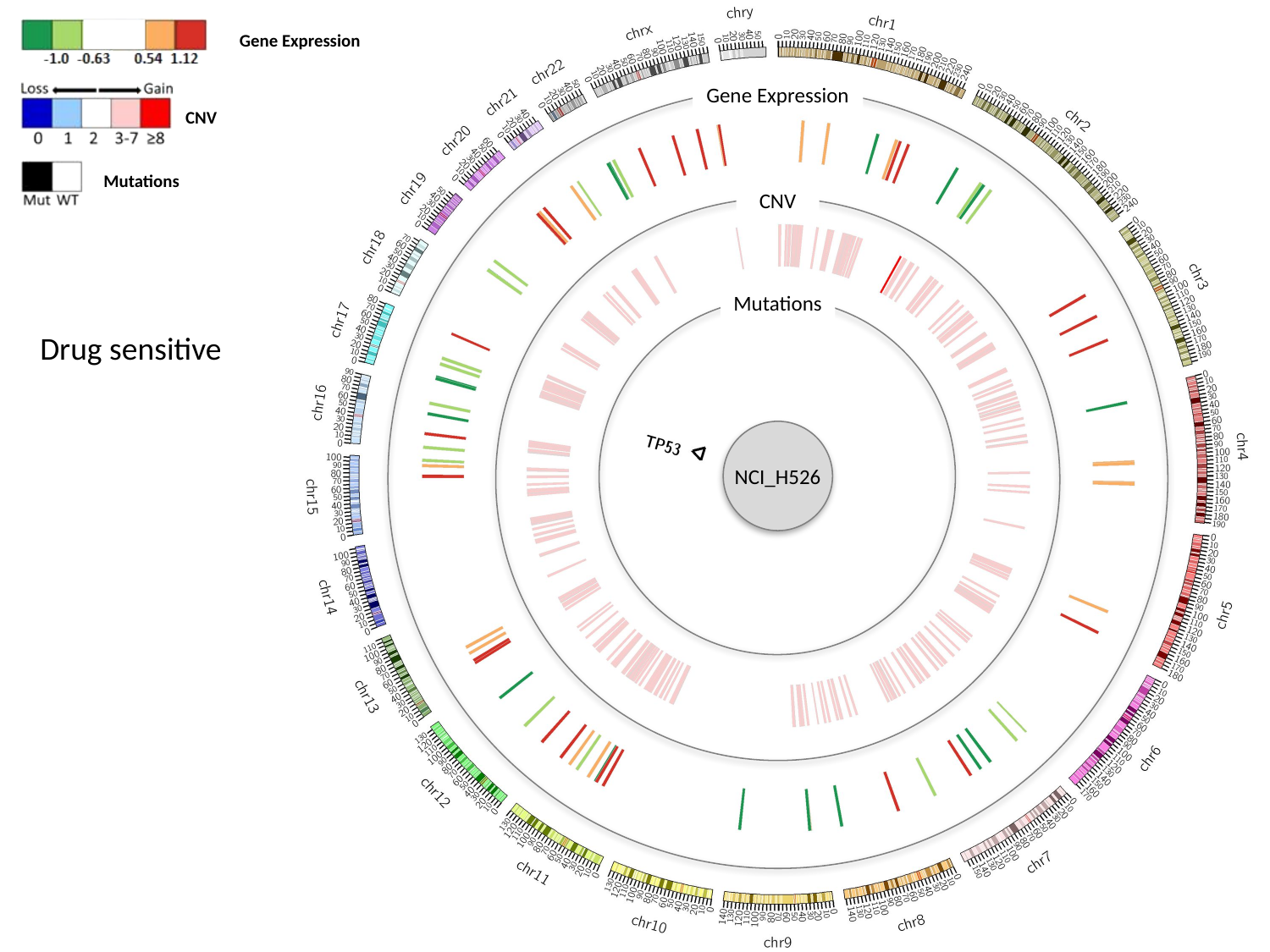

Gene Expression
CNV
Mutations
NCI_H526
Gene Expression
CNV
Mutations
Drug sensitive

## Slide 4
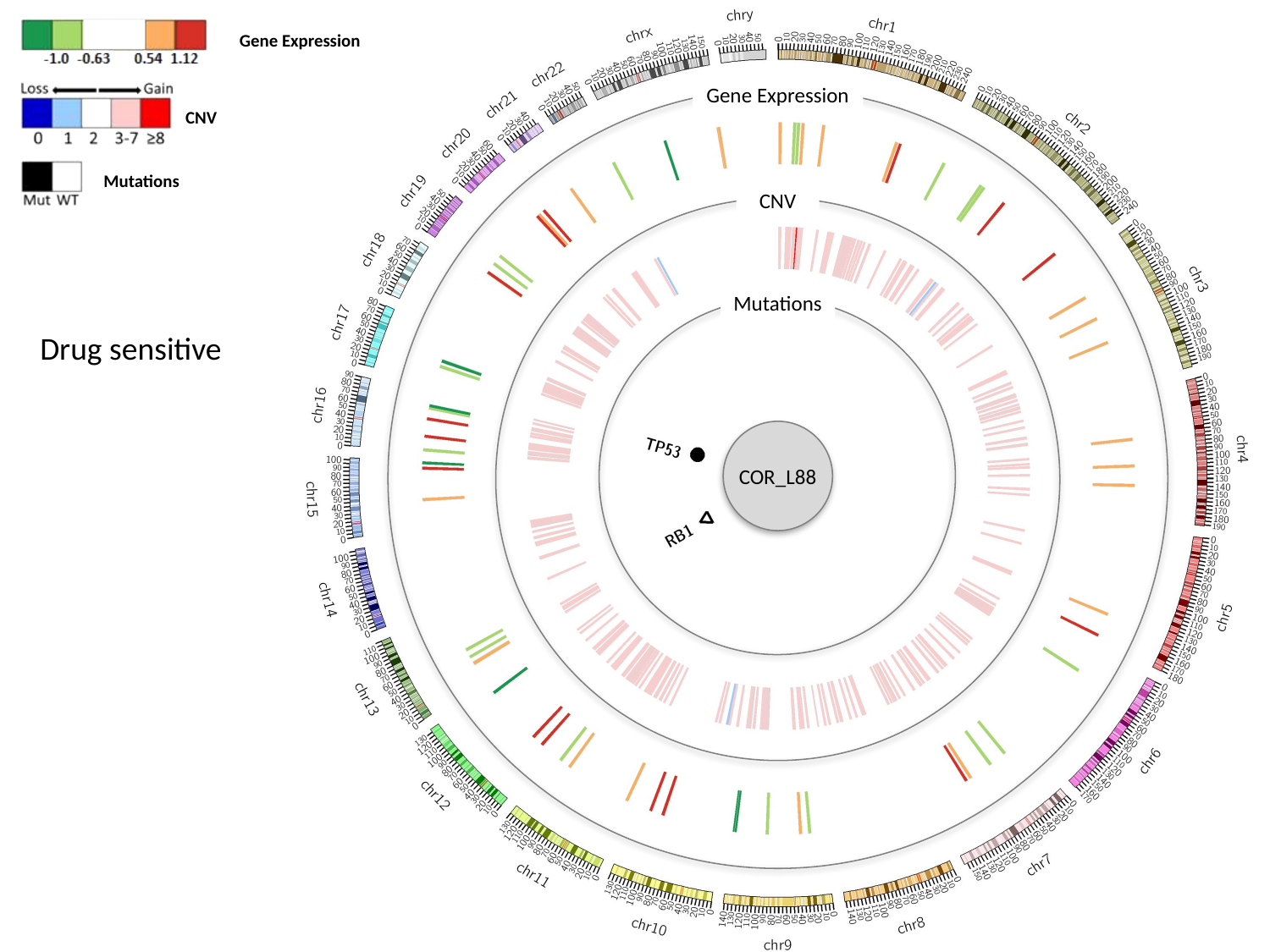

Gene Expression
CNV
Mutations
COR_L88
Gene Expression
CNV
Mutations
Drug sensitive

## Slide 5
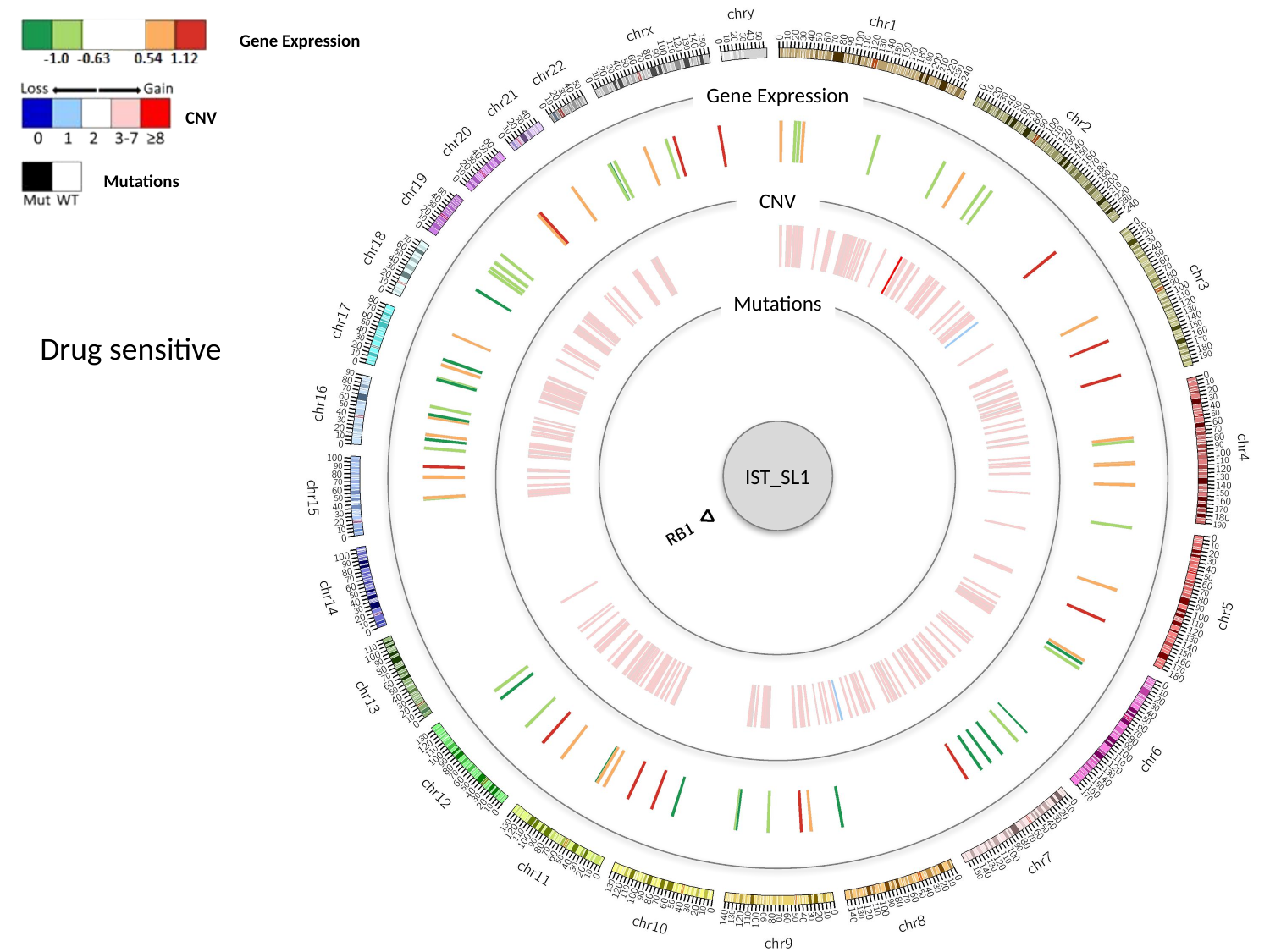

Gene Expression
CNV
Mutations
IST_SL1
Gene Expression
CNV
Mutations
Drug sensitive

## Slide 6
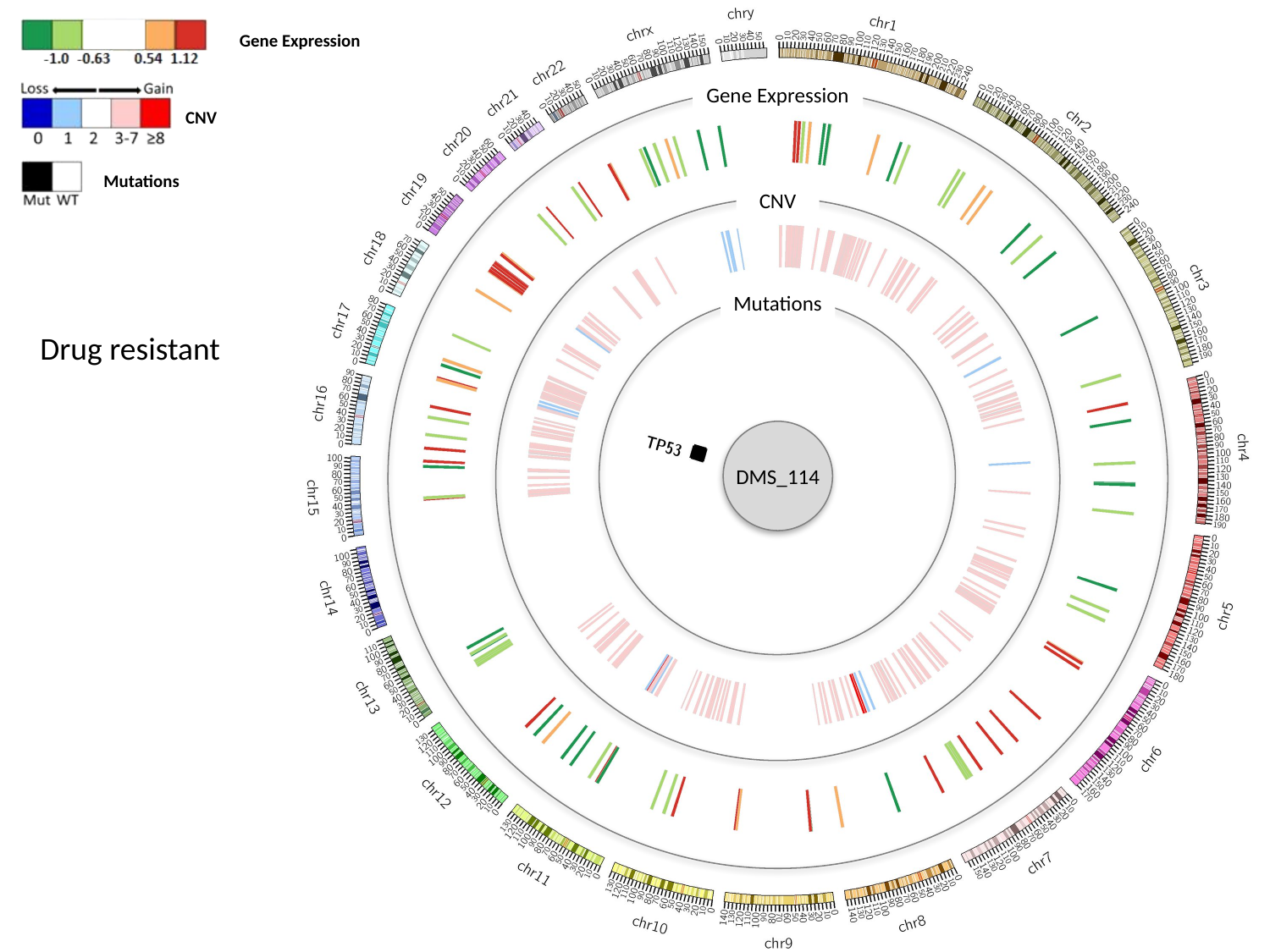

Gene Expression
CNV
Mutations
DMS_114
Gene Expression
CNV
Mutations
Drug resistant

## Slide 7
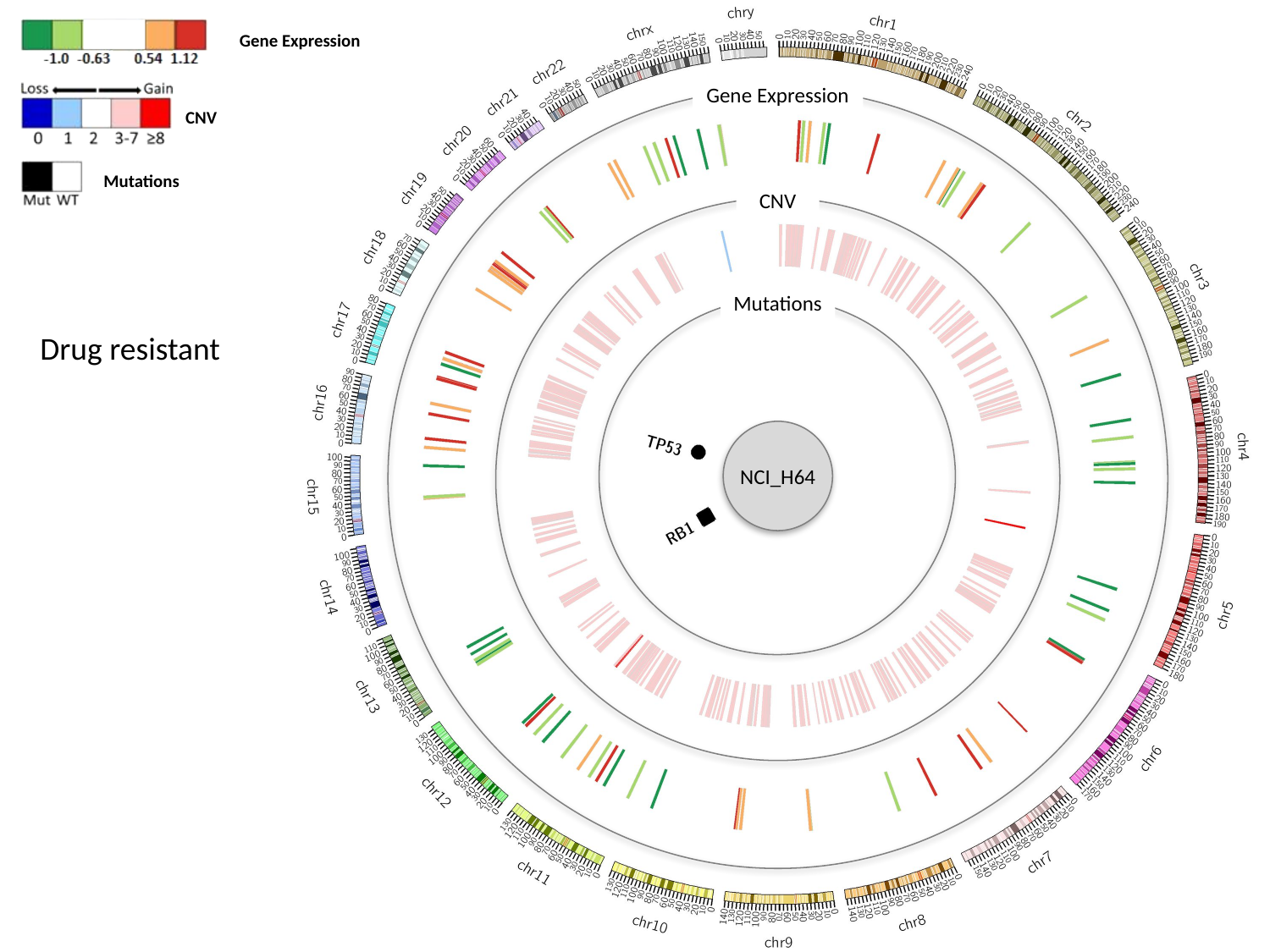

Gene Expression
CNV
Mutations
NCI_H64
Gene Expression
CNV
Mutations
Drug resistant

## Slide 8
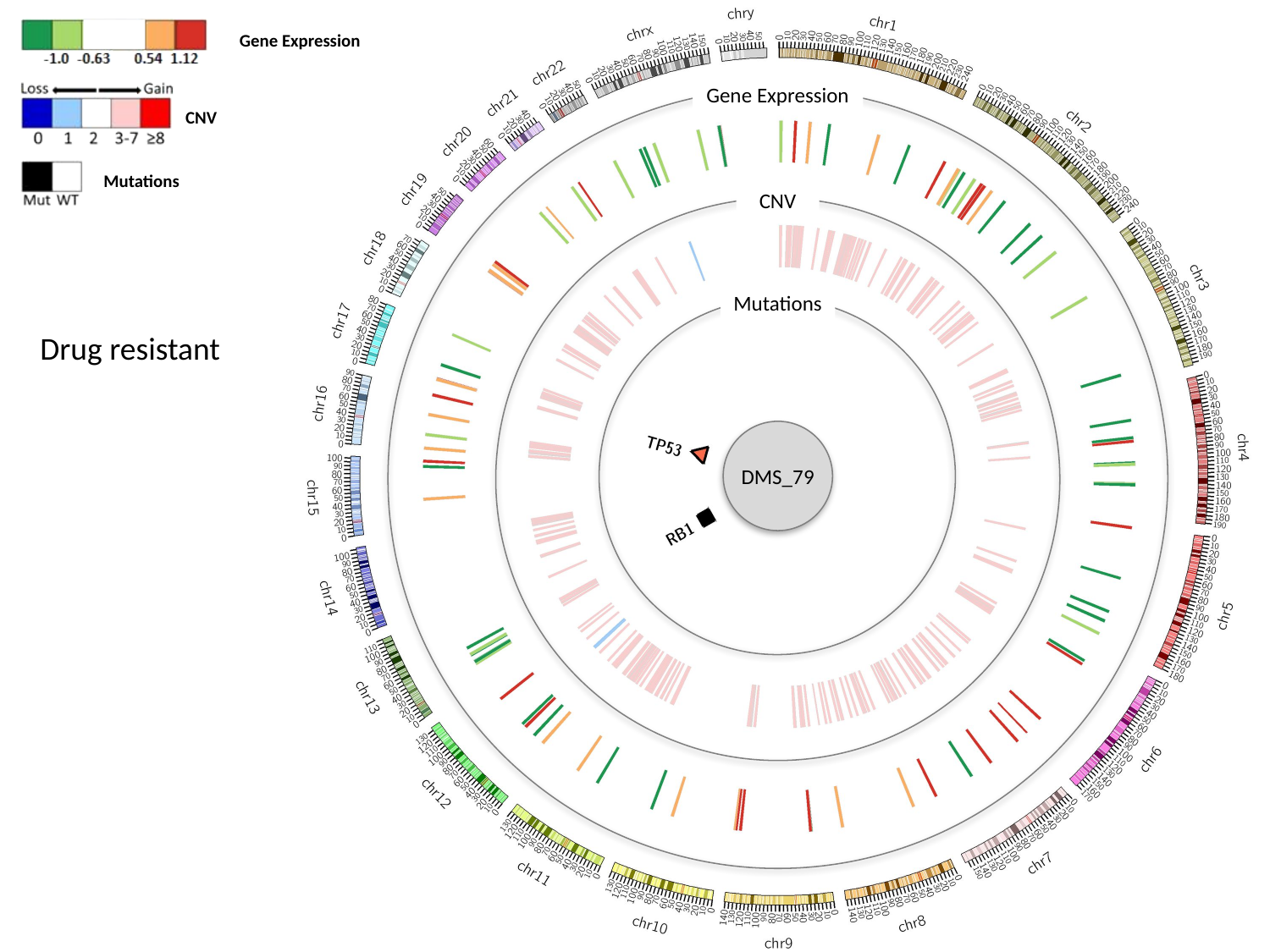

Gene Expression
CNV
Mutations
DMS_79
Gene Expression
CNV
Mutations
Drug resistant

## Slide 9
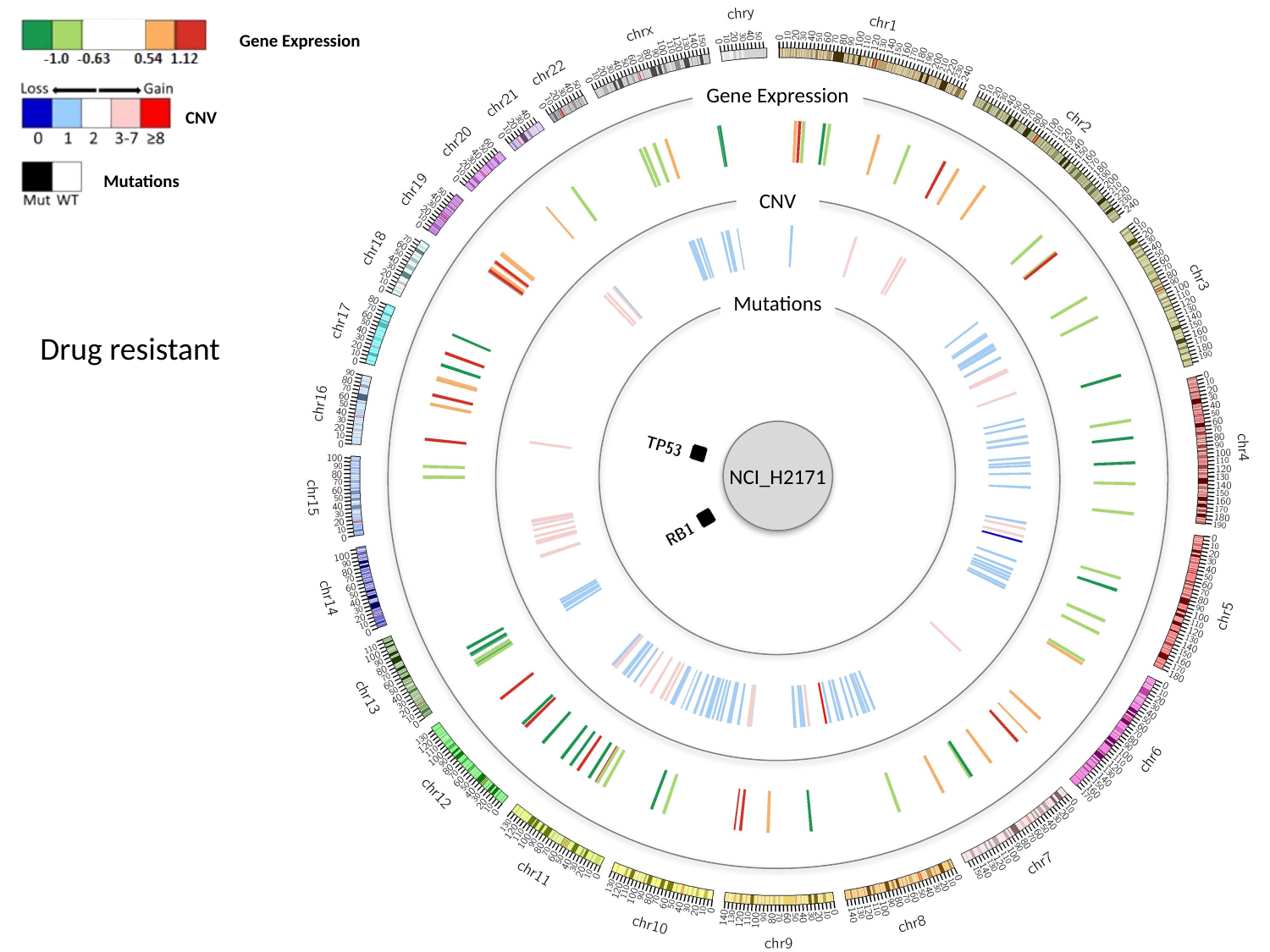

Gene Expression
CNV
Mutations
NCI_H2171
Gene Expression
CNV
Mutations
Drug resistant

## Slide 10
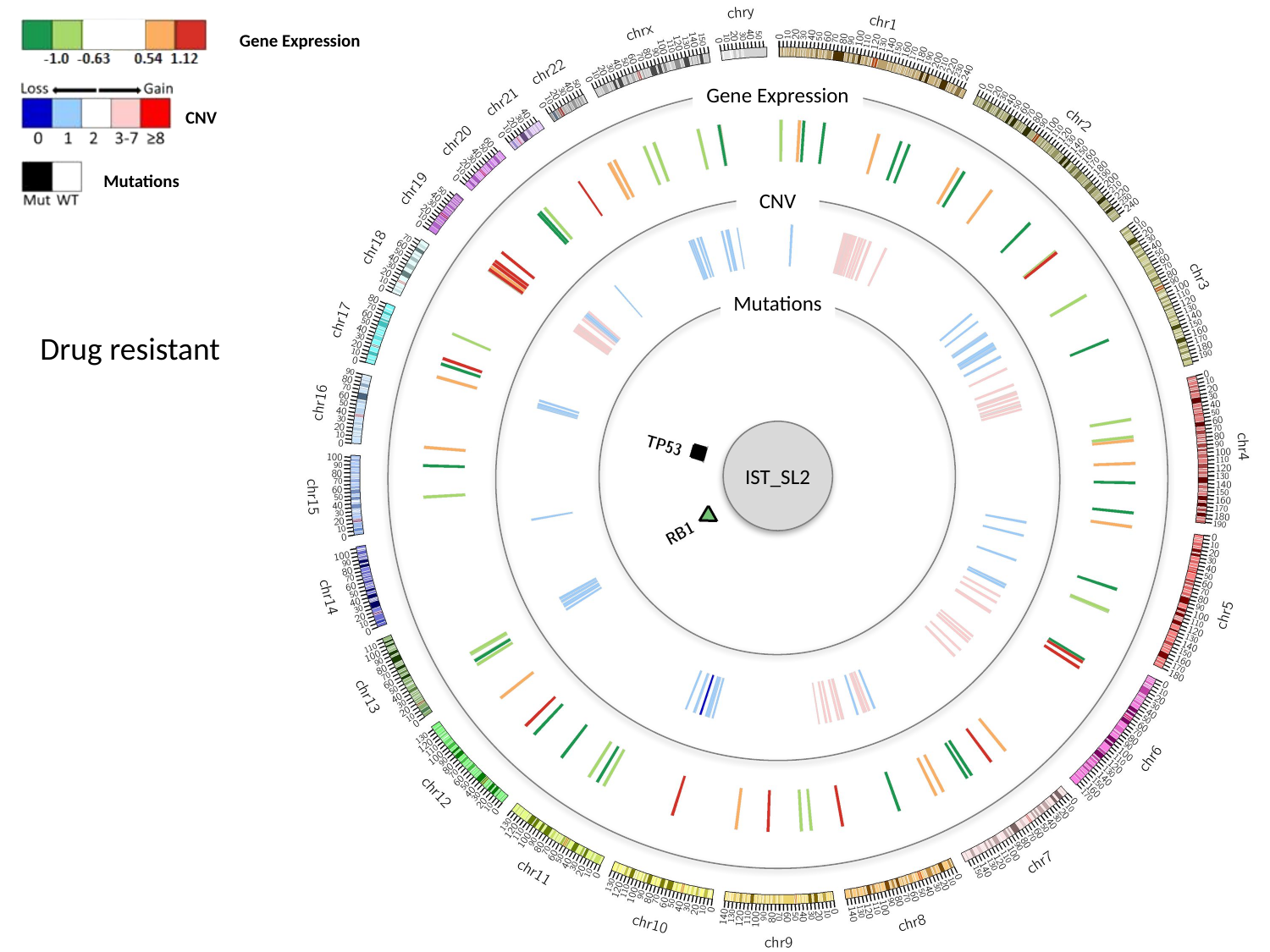

Gene Expression
CNV
Mutations
IST_SL2
Gene Expression
CNV
Mutations
Drug resistant
