# Supplementary figures and images for: Pharmacogenomic Approach to Identify Drug Sensitivity in Small-Cell Lung Cancer
Source: PLoS One. 2014 Sep 8;9(9):e106784. doi: 10.1371/journal.pone.0106784 (PMC4157793; doi:10.1371/journal.pone.0106784)

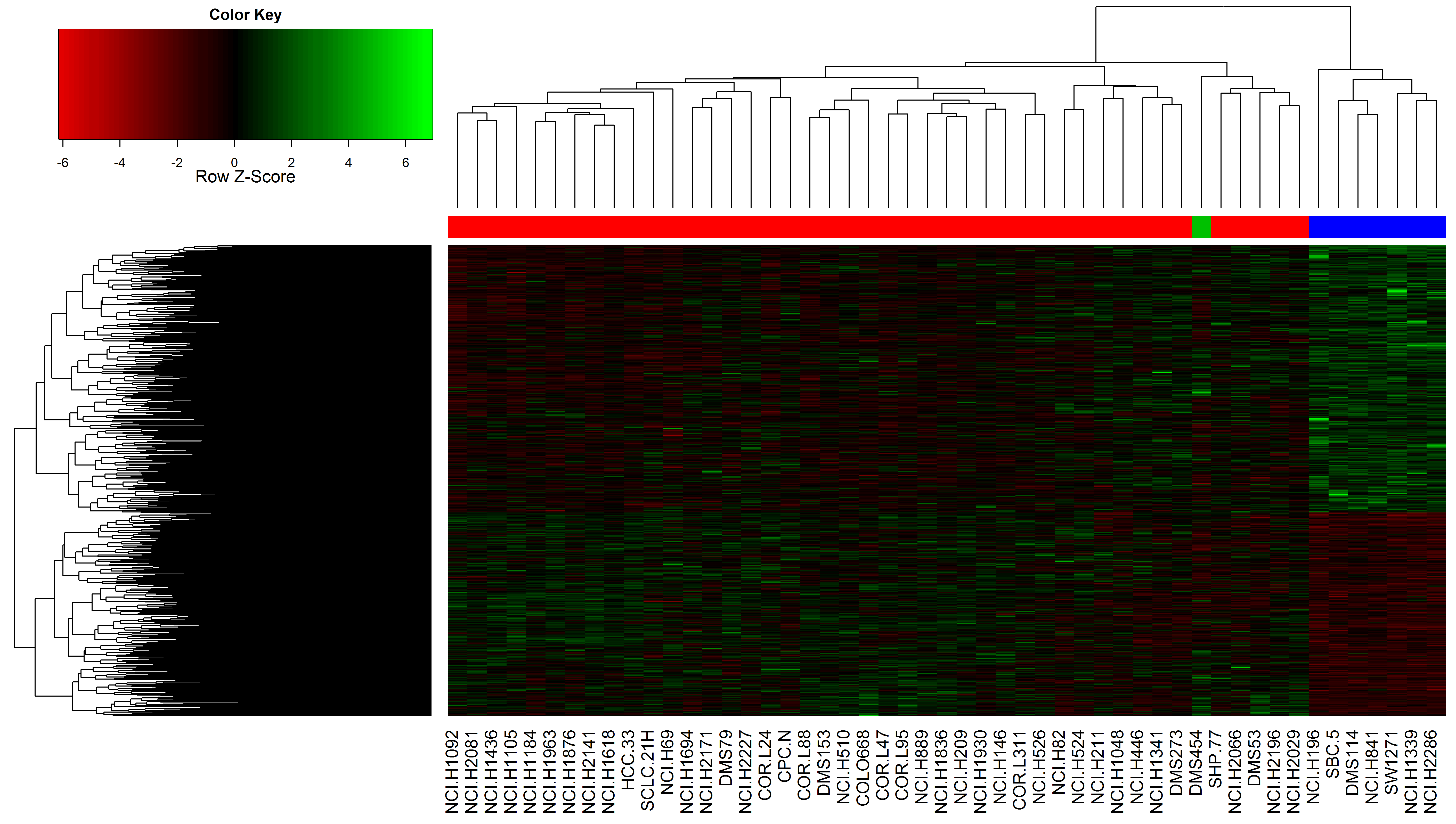

Supplement: Figure S4 — Unsupervised clustering of SCLC cells by gene expression using the CCLE dataset. Unsupervised consensus clustering was performed using the all 53 cell lines (only 51 gene expression available) and showed that 3 clusters was optimal for this dataset. With this assignment, we performed non-parametric one way ANOVA (Kruskal-Wallis) test on those 3 clusters and obtained 4749 significant genes. We then generated the heatmap with those significant genes. (TIF) [file pone.0106784.s004.tif]
