# Supplementary material for: Pharmacogenomic Approach to Identify Drug Sensitivity in Small-Cell Lung Cancer
Source: PLoS One. 2014 Sep 8;9(9):e106784. doi: 10.1371/journal.pone.0106784 (PMC4157793; doi:10.1371/journal.pone.0106784)
Supplement: Table S1 — Numerical data for drug efficacy determined in the CGP study. The 25%, 50% and 75% quantiles for all 92 drugs used to construct the boxplot in Figure 1 are listed. The outlier cell lines with IC50s <4 µM are listed to the right (IC50s of outliers in parentheses in µM). (DOC) [file pone.0106784.s005.doc]

**Table S1: The 25%, 50% and 75% quantiles and <4 μM outliers for all 92 drugs and all cell lines in the CGP dataset (IC50s of outliers in parentheses):**

| **Drug** | **25%** | **50%** | **75%** | **Outlier cell lines** |
| --- | --- | --- | --- | --- |
| Erlotinib | 8 | 8 | 8 |  |
| Rapamycin | 0.81 | 3.65 | 8 |  |
| Sunitinib | 8 | 8 | 8 | MS-1 (3.84) |
| PHA.665752 | 8 | 8 | 8 |  |
| MG.132 | 8 | 8 | 8 | IST-SL1 (0.64),NCI-H128 (1.36),NCI-H2171 (2.21) |
| Paclitaxel | 0.07 | 0.20 | 1.65 |  |
| Cyclopamine | 8 | 8 | 8 |  |
| AZ628 | 8 | 8 | 8 | ISL-SL2 (1.64),LB647-SCLC (3.68),NCI-H64 (1.60) |
| Sorafenib | 8 | 8 | 8 | NCI-H2171 (2.40) |
| VX.680 | 4.12 | 8 | 8 |  |
| Imatinib | 8 | 8 | 8 |  |
| NVP.TAE684 | 6.02 | 8 | 8 | LU-134-A (0.48),NCI-H187 (1.70),NCI-H526 (2.19) |
| PF.02341066 | 8 | 8 | 8 |  |
| AZD.0530 | 8 | 8 | 8 |  |
| S.Trityl.L.cysteine | 4.53 | 7.97 | 8 |  |
| Z.LLNle.CHO | 8 | 8 | 8 | IST-SL1 (2.53) |
| Dasatinib | 8 | 8 | 8 | NCI-H446 (0.63),SBC-1 (1.58) |
| GNF.2 | 8 | 8 | 8 | SBC-1 (1.00) |
| CGP.60474 | 0.20 | 0.39 | 1.77 |  |
| CGP.082996 | 8 | 8 | 8 |  |
| A.770041 | 8 | 8 | 8 |  |
| WH.4.023 | 8 | 8 | 8 |  |
| WZ.1.84 | 8 | 8 | 8 |  |
| BI.2536 | 0.13 | 0.46 | 2.64 |  |
| BMS.536924 | 8 | 8 | 8 | COLO-668 (1.19),NCI-H187 (2.34) |
| BMS.509744 | 8 | 8 | 8 |  |
| CMK | 8 | 8 | 8 | NCI-H128 (3.09) |
| Pyrimethamine | 8 | 8 | 8 |  |
| JW.7.52.1 | 0.49 | 1.19 | 8 |  |
| A.443654 | 0.49 | 0.75 | 6.25 |  |
| GW843682X | 0.07 | 0.33 | 6.39 |  |
| MS.275 | 1.16 | 2.61 | 8 |  |
| Parthenolide | 8 | 8 | 8 |  |
| KIN001.135 | 8 | 8 | 8 |  |
| Bortezomib | 0.01 | 0.04 | 0.35 |  |
| XMD8.85 | 8 | 8 | 8 | DMS-114 (2.41),NCI-H64 (2.20) |
| Roscovitine | 8 | 8 | 8 |  |
| Salubrinal | 8 | 8 | 8 |  |
| Lapatinib | 8 | 8 | 8 |  |
| GSK269962A | 8 | 8 | 8 | IST-SL1 (2.69),NCI-H64 (1.30) |
| Metformin | 8 | 8 | 8 |  |
| AICAR | 8 | 8 | 8 |  |
| Camptothecin | 0.002 | 0.03 | 0.27 |  |
| Vinblastine | 0.003 | 0.008 | 0.18 |  |
| Cisplatin | 5.97 | 8 | 8 | NCI-H1092 (2.06) |
| Cytarabine | 1.83 | 3.337 | 8 |  |
| Docetaxel | 0.002 | 0.006 | 0.03 |  |
| Methotrexate | 0.08 | 0.64 | 4.25 |  |
| ATRA | 8 | 8 | 8 |  |
| Gefitinib | 1.22 | 7.93 | 8 |  |
| ABT.263 | 0.41 | 5.79 | 8 |  |
| Vorinostat | 2.05 | 2.32 | 6.43 |  |
| Nilotinib | 8 | 8 | 8 |  |
| RDEA119 | 8 | 8 | 8 |  |
| CI.1040 | 8 | 8 | 8 |  |
| Temsirolimus | 0.08 | 0.52 | 6.31 |  |
| AZD.2281 | 7.59 | 8 | 8 | NCI-H1092 (3.87),NCI-H64 (3.83) |
| ABT.888 | 8 | 8 | 8 |  |
| Bosutinib | 8 | 8 | 8 | NCI-H1092 (1.99) |
| Lenalidomide | 8 | 8 | 8 |  |
| Axitinib | 4.96 | 8 | 8 |  |
| AZD7762 | 0.64 | 1.15 | 4.33 |  |
| GW.441756 | 8 | 8 | 8 |  |
| CEP.701 | 0.56 | 3.22 | 8 |  |
| SB.216763 | 8 | 8 | 8 | NCI-H1092 (0.05) |
| X17.AAG | 0.62 | 2.11 | 4.94 |  |
| VX.702 | 7.72 | 8 | 8 | NCI-H1694 (3.89),NCI-H64 (2.93) |
| AMG.706 | 8 | 8 | 8 |  |
| KU.55933 | 8 | 8 | 8 |  |
| Elesclomol | 0.04 | 0.28 | 1.98 |  |
| BIBW2992 | 8 | 8 | 8 | NCI-H1092 (0.078),NCI-H1694 (1.54) |
| GDC.0449 | 8 | 8 | 8 |  |
| PLX4720 | 8 | 8 | 8 |  |
| BX.795 | 6.47 | 8 | 8 | NCI-H2171 (2.32),NCI-H378 (3.43) |
| NU.7441 | 6.98 | 8 | 8 | NCI-H1092 (0.28) |
| SL.0101.1 | 8 | 8 | 8 |  |
| BI.D1870 | 3.82 | 8 | 8 |  |
| BIRB.0796 | 8 | 8 | 8 |  |
| JNK.Inhibitor.VIII | 8 | 8 | 8 |  |
| X681640 | 1.91 | 8 | 8 |  |
| Nutlin.3a | 8 | 8 | 8 |  |
| PD.173074 | 8 | 8 | 8 | DMS-153 (3.20) |
| ZM.447439 | 6.50 | 8 | 8 | NCI-H2171 (1.32) |
| RO.3306 | 8 | 8 | 8 |  |
| MK.2206 | 6.60 | 8 | 8 | DMS-114 (1.52),NCI-H378 (3.88) |
| PD.0332991 | 8 | 8 | 8 |  |
| NVP.BEZ235 | 0.09 | 0.43 | 1.43 |  |
| GDC0941 | 4.97 | 8 | 8 |  |
| AZD8055 | 1.23 | 4.02 | 8 |  |
| PD.0325901 | 5.33 | 8 | 8 | NCI-H2107 (1.13) |
| SB590885 | 8 | 8 | 8 |  |
| AZD6244 | 8 | 8 | 8 |  |
