# Supplementary material for: Pharmacogenomic Approach to Identify Drug Sensitivity in Small-Cell Lung Cancer
Source: PLoS One. 2014 Sep 8;9(9):e106784. doi: 10.1371/journal.pone.0106784 (PMC4157793; doi:10.1371/journal.pone.0106784)
Supplement: Table S2 — Numerical data for drug efficacy determined in the CCLE study. The 25%, 50% and 75% quantiles for all 24 drugs used to construct the boxplot in Figure S1 are listed. The outlier cell lines with IC50s <4 µM are listed to the right (IC50s of outliers in parentheses in µM). (DOC) [file pone.0106784.s006.doc]

**Table S2: The 25%, 50% and 75% quantiles and < 4μM outliers for all 24 drugs and all cell lines in the CCLE dataset (IC50s of outliers in parentheses):**

| **Drug** | **25%** | **50%** | **75%** | **Outlier cell lines** |
| --- | --- | --- | --- | --- |
| 17-AAG | 0.042 | 0.19 | 0.50 |  |
| AEW541 | 3.24 | 8 | 8 |  |
| AZD0530 | 6.85 | 8 | 8 | NCI-H1048 (3.12), NCI-H2286 (3.63) |
| AZD6244 | 8 | 8 | 8 | SW 1271 (1.71) |
| Erlotinib | 8 | 8 | 8 |  |
| Irinotecan | 0.06 | 0.16 | 0.20 |  |
| L-685458 | 7.98 | 8 | 8 | NCI-H211 (3.81) |
| Lapatinib | 8 | 8 | 8 |  |
| LBW242 | 8 | 8 | 8 |  |
| Nilotinib | 6.92 | 8 | 8 | NCI-H2286 (2.39) |
| Nutlin-3 | 8 | 8 | 8 |  |
| Paclitaxel | 0.01 | 0.01 | 0.13 |  |
| Panobinostat | 0.02 | 0.05 | 0.07 |  |
| PD-0325901 | 4.99 | 8 | 8 |  |
| PD-0332991 | 8 | 8 | 8 | NCI-H2286 (1.43) |
| PF2341066 | 4.12 | 7.07 | 8 |  |
| PHA-665752 | 8 | 8 | 8 |  |
| PLX4720 | 8 | 8 | 8 |  |
| RAF265 | 2.92 | 4.18 | 8 |  |
| Sorafenib | 7.16 | 8 | 8 |  |
| TAE684 | 1.44 | 5.20 | 8 |  |
| TKI258 | 3.69 | 6.60 | 8 |  |
| Topotecan | 0.06 | 0.14 | 0.19 |  |
| ZD-6474 | 8 | 8 | 8 | NCI-H1048 (2.66) |
